# Dynamic Distribution of the Gut Microbiota and the Relationship with Apparent Crude Fiber Digestibility and Growth Stages in Pigs

**DOI:** 10.1038/srep09938

**Published:** 2015-04-21

**Authors:** Qing Niu, Pinghua Li, Shuaishuai Hao, Yeqiu Zhang, Sung Woo Kim, Huizhi Li, Xiang Ma, Shuo Gao, Lichun He, WangJun Wu, Xuegen Huang, Jindi Hua, Bo Zhou, Ruihua Huang

**Affiliations:** 1 Institute of Swine Science, Nanjing Agricultural University, Nanjing, 210095, China; 2 Huaian Academy of Nanjing Agricultural University, Huaian, 223005, China; 3 Department of Animal Science, North Carolina State University, Raleigh, North Carolina, 27695, United States of America; 4 Sutai Pig Breeding Center, Suzhou, 215000, China

## Abstract

The gut microbiota plays an important role in nutrient digestibility in animals. To examine changes in the pig gut microbiota across growth stages and its effects on nutrient digestion, the gut microbiota population in pigs at 28 days (before weaning), and 60, 90, and 150 days of age was assessed by 16S rDNA gene sequencing. The apparent digestibility of crude fiber (CF), neutral detergent fiber (NDF), acid detergent fiber (ADF), crude protein (CP) and ether extract (EE) was also assessed in these pigs. A total of 19,875 operational taxonomic units (OTUs) were identified from all samples. Both bacterial abundance and diversity increased with age. A total of 22 phyla and 249 genera were identified from all fecal samples; *Firmicutes* and *Bacteroidetes* were the most dominant phyla in all samples. With increasing age, the proportion of *TM7* and *Tenericutes* increased, whereas the proportion of *Lentisphaerae* and *Synergistetes* decreased. The abundance of 36 genera varied with age, and the apparent digestibility of CF increased with age. Three phyla, *Proteobacteria*, *Tenericutes* and *TM7*, and 11 genera, including *Anaeroplasma*, *Campylobacter*, and *Clostridium*, were correlated with apparent CF digestibility.

The large and diverse microbial population contained in the mammalian gastrointestinal tract plays an important role in health and nutrient digestion^1^,^2^,^3^. The distal gut harbors the majority of human gut microorganisms, with approximately 10^12^ microbes per milliliter of luminal contents, and there are at least 500–1,000 species reside in the adult human intestine^4^,^5^,^6^,^7^. The microbes of animals live in intimate contact with each other, and a set of mutualistic or even symbiotic relationships has developed between the host and microbes^8^. Gut microbiota participate in digestion, absorption, and metabolism of the host through the absorption of nutrients and the expulsion of metabolic wastes from the host^7^,^9^,^10^. The gut microbiota is dynamic and varies according to time, age and many other factors^2^. Because bacteria reproduce within the host and change with age^1^,^8^,^9^, it is important to study the community structure of gut bacteria and to recognize the functions of the gut microbiota in contact with the host. Pigs provide products for human consumption on a large scale, and serve as an important animal model for human diseases, including obesity, diabetes and metabolic disorders, because of the similarity between pigs and humans in physiology, organ development and disease progression^7^,^11^. Elucidating changes in the pig gut microbiota and the correlation of these changes with nutrient digestibility across growth stages not only is essential for determining the function of the microbiota in nutrient digestion in pigs but also is beneficial for understanding how the gut microbiota of humans is related to complex traits such as obesity and metabolic disorders. However, information regarding how the pig gut microbiota varies across growth stages is limited. Likewise, little is known regarding how the pig gut microbiota contributes to swine nutrient digestion across growth stages. Dietary fiber is mainly degraded by the gut microbiota, and bacterial fermentation end products in the colon provide pigs with 5–20% of their total energy^10^,^12^.

We hypothesized that the growth stage of pigs would affect the gut microbiota and apparent crude fiber (CF) digestibility. The objective of this study was to investigate changes in the gut microbiota and apparent CF digestibility in pigs at different growth stages, as well as to elucidate the correlation between the microbiota and apparent CF digestibility in pigs.

## Results

### DNA sequence data and bacterial community structure

A total of 5,737,794 paired-end 250-bp reads were acquired. The total read length was 5.96 gigabases (GB), and the average read length per sample was 0.18 GB, with 1,532,635, 1,532,635, 1,364,816 and 1,307,708 raw reads at 28, 60, 90 and 150 days of age, respectively (Table S1).

**Figure 1:**
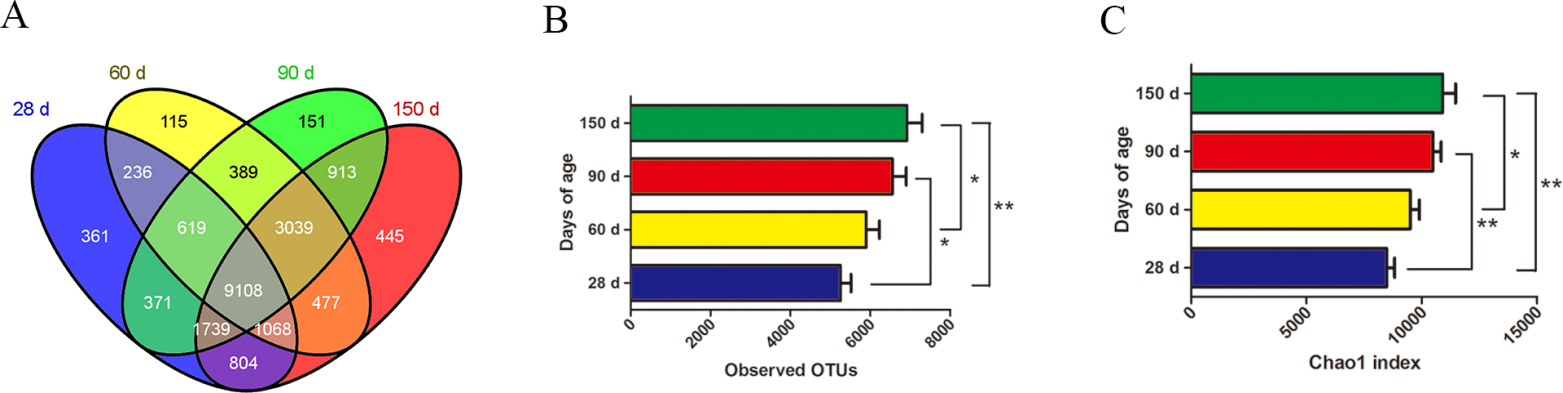
Comparison of the OTUs in the four groups. The number of observed OTUs sharing ≥97% nucleotide sequence identity. **(A)** A Venn diagram was generated to describe the common and unique OTUs among the 4 groups. **(B)** From the numbers of OTUs in the four groups, we found that bacterial diversity increased with age. **(C)** The bacterial abundance is reflected in the Chao1 index; the Chao1 index was significantly different between the four groups (*P<0.05, **P<0.01), indicating that bacterial abundance increased with age.

After initial quality control, 3,928,659 high quality sequences were obtained. On average, 119,050 sequences were obtained per sample. Based on 97% species similarity, 14,306, 15,051, 16,329 and 17,593 operational taxonomic units (OTUs) were separately obtained from samples at 28, 60, 90 and 150 days of age (Table S1). A total of 19,875 OTUs were identified from all fecal samples, with 9,108 of those existing in all groups defined as core OTUs (Fig. 1A). The core OTUs comprised approximately 46% of the total OTUs while 361, 115, 151 and 445 OTUs were uniquely identified at 28, 60, 90 and 150 days of age, respectively.

Good's coverage was 98.0, 97.2, 97.2 and 97.3% for 28, 60, 90 and 150 days of age, respectively, suggesting that the present study captured the dominant phylotypes. Both the abundance (R^2^ = 0.30, *P*<0.01) and diversity (R^2^ = 0.34, *P*<0.01) of the gut microbiota were correlated with the age of the pigs (Fig. 1B, C). The principal coordinates analysis (PCoA) of UniFrac distance matrices indicated that the variation in our data set was primarily explained by the growth stages. The 28-d group microbiota clustered separately from the microbiota of the other three groups along principal coordinate 1 (Fig. S1A, C). In addition, the 150-d group microbiota clustered separately from the microbiota of the other three groups along principal coordinate 3 (Fig. S1B, C).

**Figure 2:**
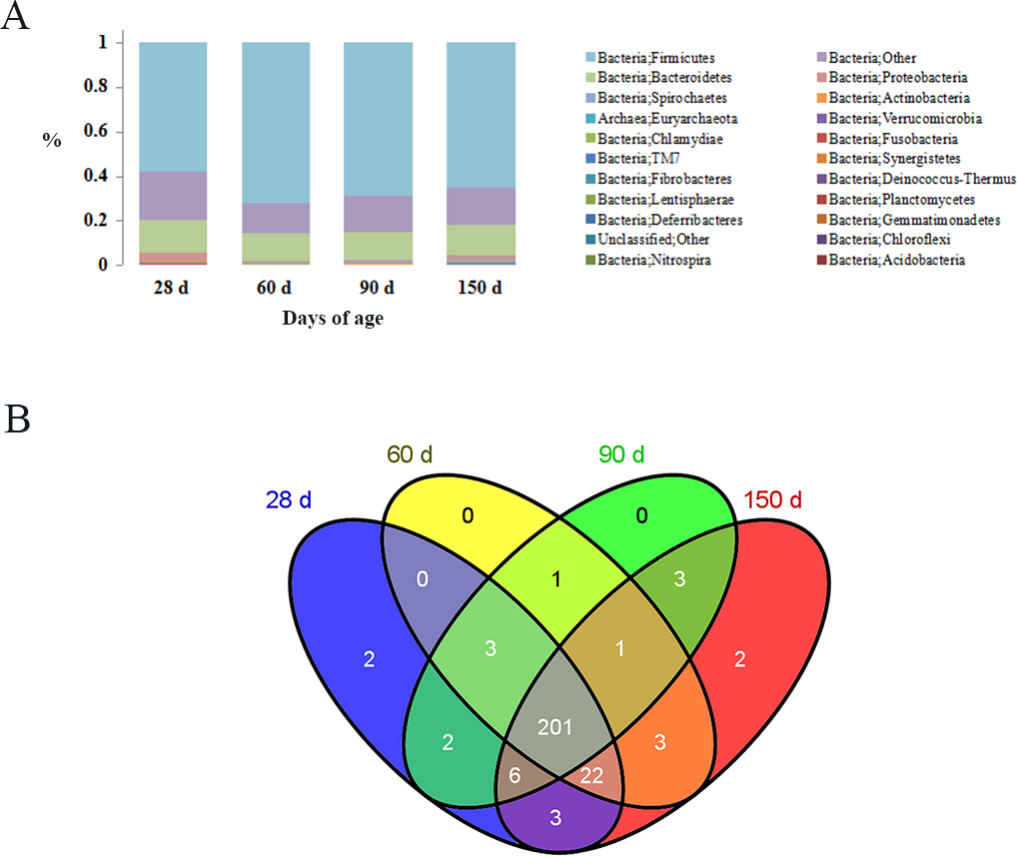
Phyla distribution of gut flora and a Venn diagram of the genera. Distribution of the phyla as a percentage of the total number of identified 16S rDNA sequences in individual groups (A). A Venn diagram was generated to compare genera between the groups at the same time points and to depict genera that were unique to the 4 groups (B).

**Figure 3:**
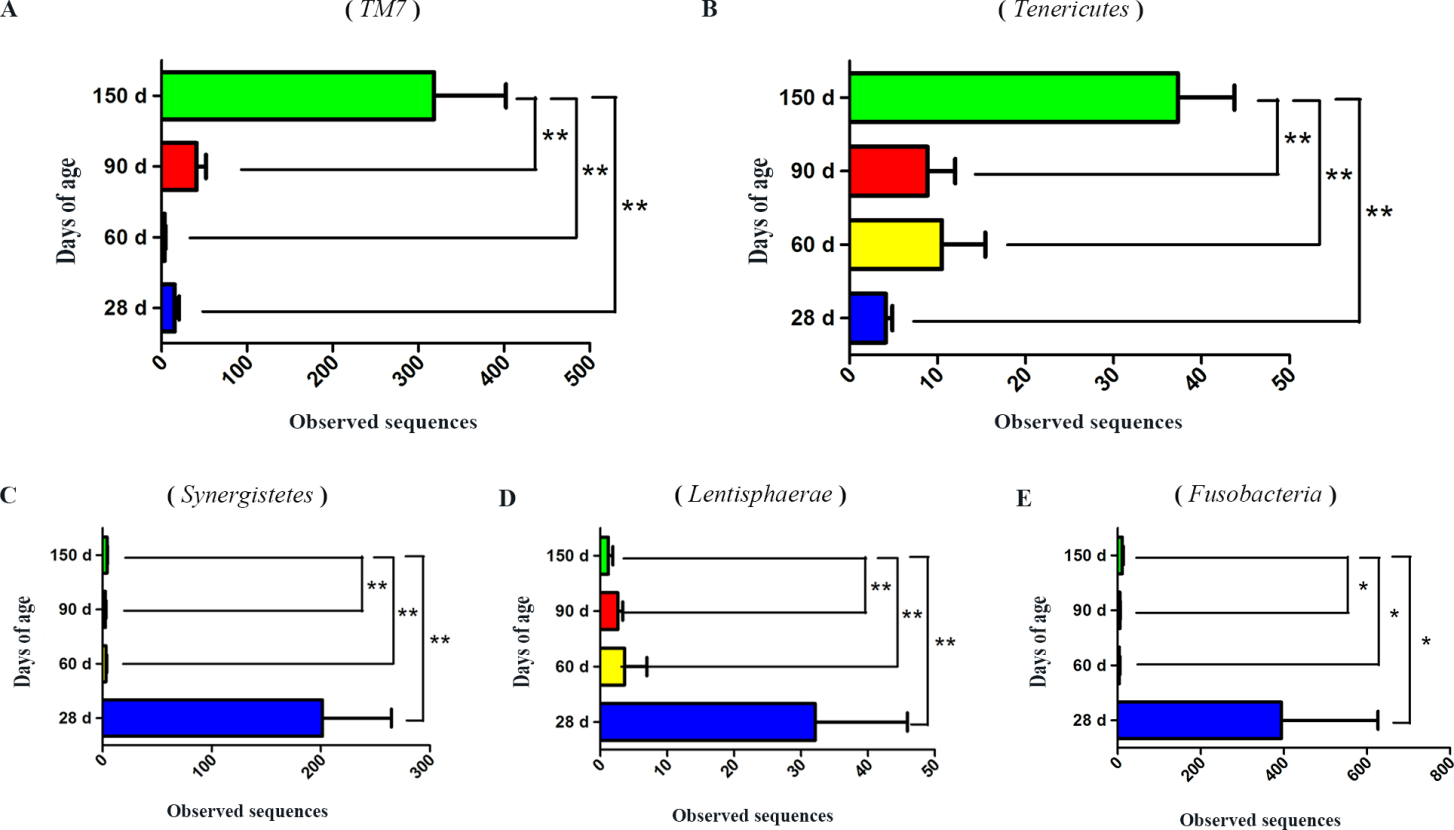
The bacterial abundances of 5 distinct phyla significantly differ among the four groups.

The results shown in Fig. 2A describe the distribution of DNA sequences into phyla. A total of 22 phyla were shared by the four growth-stage groups, as follows: *Acidobacteria*, *Actinobacteria*, *Bacteroidetes*, *Chlamydiae*, *Chloroflexi*, *Cyanobacteria, Deferribacteres*, *Deinococcus-Thermus*, *Euryarchaeota*, *Fibrobacteres*, *Firmicutes*, *Fusobacteria*, *Gemmatimonadetes*, *Lentisphaerae*, *Nitrospira*, *Planctomycetes*, *Proteobacteria*, *Spirochaetes, Synergistetes, Tenericutes, TM7* and *Verrucomicrobia*. *Firmicutes* and *Bacteroidetes* were the most dominant among the 22 phyla (*P*<0.01) in the samples, regardless of age, and comprised more than 79% of the total sequences. The bacterial abundances of distinct phyla differed in the four groups. *Firmicutes* (*P*<0.01) was the most predominant phylum at 28 days of age, accounting for approximately 58% of the sequences. At 60, 90 and 150 days of age, a higher percentage (72, 69 and 65%, respectively) of the sequences was assigned to *Firmicutes*. *Bacteroidetes* (*P*<0.01) was the second largest phylum in all groups, comprising approximately 15, 12, 13 and 13% in the 28-d, 60-d, 90-d and 150-d groups, respectively. Significant differences in the bacterial abundance in 5 of the 22 phyla were found in the four groups (Fig. 3A-E). Moreover, the abundance in 4 of the 5 phyla changed with age (Table S2). The proportion of bacteria in *TM7* (Fig. 3A) and *Tenericutes* (Fig. 3B) increased (*P*<0.01) as the pigs aged, whereas the proportion of bacteria in *Synergistetes* (*P*<0.01) (Fig. 3C) and *Lentisphaerae* (*P*<0.05) (Fig. 3D) decreased. The proportion of sequences that could not be assigned to a phylum using the Ribosomal Database Project (RDP) classifier was 17.4% in the fecal samples.

At the genus level, a total of 249 genera were identified from all samples, regardless of age (Fig. 2B). The 16 most abundant genera, containing more than 84% of the total sequences, were *Lactobacillus*, *Subdoligranulum*, *Roseburia*, *Oscillibacter*, *Eubacterium*, *Dorea*, *Streptococcus*, *Clostridium*, *Megasphaera*, *Escherichia/Shigella*, *Oribacterium*, *Blautia*, *Faecalibacterium*, *Coprococcus*, *Ruminococcus* and *Treponema*. One genus, *Escherichia/Shigella*, is a member of the phylum *Proteobacteria,* and *Treponema* belongs to the phylum *Spirochaetes*. The other 14 genera belong to the phylum *Firmicutes*. Among them, *Lactobacillus*, *Gemmiger* and *Roseburia* were the most predominant genera, accounting for 15, 13 and 11% of total sequences, respectively. The number of genera in the 28-d, 60-d, 90-d and 150-d groups was 239, 231, 217 and 241, respectively. A total of 201 genera were shared by the four different groups. *Leptospirillum* and *Leuconostoc* were unique to the 28-d group, and *Solitalea* and *Porphyrobacter* were specific to the 150-d group (Fig. 2B). The bacterial abundance of 36 genera changed as the pigs aged; 26 increased, whereas 10 decreased (Table S3).

In addition to the phylum and genus, bacterial diversity and abundance were also analyzed by class (Table S4), order (Table S5) and family (Table S6). A total of 48, 47, 44 and 49 classes, 80, 79, 72 and 83 orders and 155, 137, 84 and 159 families were identified at 28, 60, 90 and 150 days of age, respectively. The most predominant class and family shared in the 28-d, 60-d, 90-d and 150-d groups were *Clostridia* and *Clostridiales*, respectively. The most predominant order was *Ruminococcaceae* in the 28-d group and *Lachnospiraceae* in the 60-d, 90-d and 150-d groups.

### Apparent nutrient digestibility in pigs and its correlation with the gut microbiota

The apparent digestibility of total tract CF and acid detergent fiber (ADF) increased as the pigs aged (*P*<0.01) (Table 1). At the phylum level, *Proteobacteria*, *Tenericutes* and *TM7* were positively correlated with apparent CF digestibility (*P*<0.05) (Table 2). *Proteobacteria* and *TM7* were positively correlated with apparent ADF digestibility (*P*<0.01) (Table 2). At the genus level, the bacterial abundance of *Anaeroplasma*, *Campylobacter*, *Clostridium*, *Enterococcus*, *Methanobrevibacter, Nitrosospira*, *Propionibacterium*, *Pseudobutyrivibrio*, *Robinsoniella*, *Staphylococcus* and *Treponema* was positively correlated with apparent CF digestibility (*P*<0.05) (Table 3). The bacterial abundance of *Anaeroplasma*, *Campylobacter*, *Caulobacter*, *Cloacibacillus*, *Enterococcus*, *Lactobacillus*, *Methanobrevibacter*, *Nitrosospira*, *Propionibacterium*, *Pseudomonas*, *Robinsoniella*, *Staphylococcus* and *Treponema* was positively correlated with apparent ADF digestibility (*P*<0.05) (Table 3). In addition to the phylum and genus, the correlation analysis between the apparent fiber digestibility and gut microbiota in pigs was also analyzed by class (Table S7), order (Table S8) and family (Table S9).

The apparent total tract crude protein (CP) digestibility was higher in the 90-d (*P*<0.05) and 150-d (*P*<0.01) groups than that in the 60-d group, whereas the two younger groups showed no difference (Table 1). However, this study did not find a positive correlation between the gut microbiota and apparent CP digestibility among the three groups. No significant difference was found for the apparent ether extract (EE) digestibility among the three groups (Table 1).

## Discussion

The first goal of this study was to address the question of how the pig gut microbiota change as pigs grow. So far, few reports have been published on the distribution of the gut microbiota across pig growth stages^1^,^2^,^3^,^13^,^14^.

A total of 3,928,659 high-quality sequences were obtained from samples at all ages; the average number of reads per sample was 119,050, and the read counts were greater than those in previous studies in pigs^1^,^13^,^14^,^15^. Moreover, according to Good's coverage index of each sample, the modified sequences were comprehensive enough to cover most bacterial diversity. The number of total effective OTUs in this study was higher than that in the studies of Kim HB *et al*^13^, Ye L, Zhang T^14^ and Whitehead TR and Cotta MA^17^, indicating a much higher level of diversity than reported in previous studies.

As bacterial abundance and diversity increased with age, growth stages and growth conditions were important factors affecting pig gut microbiota. In a previous study, Kim HB *et al*^13^ described the changes in bacterial microbiota composition that occurred between pigs in different age groups. Savage DC^18^ demonstrated that the gastrointestinal microbiota changed over time from birth through adulthood. Rosenberg E^19^ reported considerable species variation in the gut microbiota among healthy individuals^17^. Furthermore, the human gut microbiota undergoes maturation from birth to adulthood and is further altered with aging^20^. Moreover, considering any of the other taxonomic units, i.e., class, order and family, the bacterial diversity of the 150-d group was always higher than that of the other three groups.

Using the RDP classifier, taxon-dependent analysis revealed that *Firmicutes* and *Bacteroidetes* were the most dominant phyla, regardless of age, representing approximately 79% of the total sequences. These results were similar to what others have observed in pig and human fecal samples^1^,^13^,^17^. Kim HB *et al*^13^ reported that the bacterial communities of all samples were primarily comprised of *Firmicutes* and *Bacteroidetes*, which accounted for more than 90% of the total sequences. Lamendella R *et al*^15^ confirmed previous observations that most of the bacteria identified were in two phyla: *Firmicutes* and *Bacteroidetes*. Additionally, these two phyla comprised over 90% of the known phylogenetic categories, representing the dominant distal gut microbiota in the human intestinal tract^21^. However, the proportion of *TM7* and *Tenericutes* increased as the pigs aged, and the proportion of *Lentisphaerae*, *Synergistetes* and *Fusobacteria* decreased. Kim HB *et al*^1^ found that the proportion of bacteria in the phyla *Firmicutes* and *Spirochaetes* increased as the pigs aged, whereas the proportion of bacteria in the phyla *Bacteroidetes*, *Actinobacteria* and *Proteobacteria* decreased. The discrepancies between the present study and previous studies may have resulted from the use of pigs in different ages, environmental conditions or breeds^2^. Moreover, much deeper sequencing was performed for the samples in this study.

In the present study, the bacterial abundance of 36 genera changed as the pigs aged; 26 increased, whereas 10 decreased. Interestingly, of the 36 genera, *Lactobacillus, Pseudobutyrivibrio* and *Butyricicoccus* increased in our study but decreased in Kim HB's study^1^. The discrepancies between the present study and previous studies may also be due to the use of pigs in different ages, environmental conditions or breeds. *Leptospirillum* and *Leuconostoc* were unique to the 28-d group, and *Solitalea* and *Porphyrobacter* were specific to the 150-d group. *Leuconostoc* is an industrially important microorganism that is used as a starter culture in the manufacturing process of several fermented foods, including kimchi and sauerkraut, and has great potential for biotechnological applications such as enzyme and vaccine delivery systems^22^. *Porphyrobacter* functions to produce Bacteriochlorophyll a^23^. However, the functions of *Leptospirillum* and *Solitalea* require further study*.*

**Figure 4:**
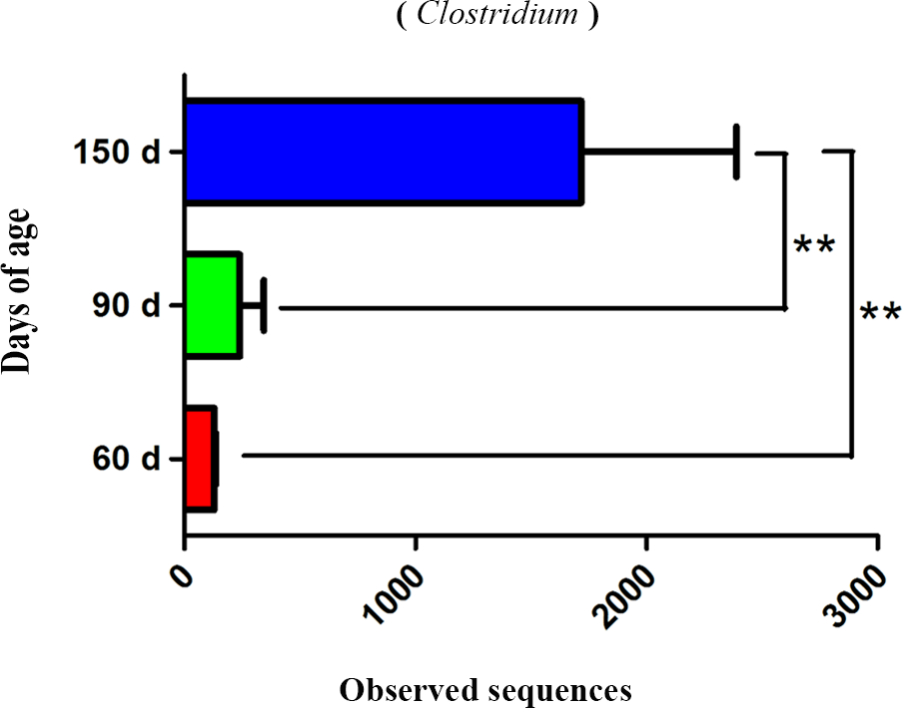
The bacterial abundance of *Clostridium* significantly differs between the four groups.

The second goal of this study was to preliminarily reveal changes in nutrient digestibility and the relationship with the abundance and diversity of the gut microbiota in pigs in different growth stages. The apparent digestibility of CF, NDF, ADF, hemicellulose, CP and EE in pig fecal samples was analyzed, except in piglets at 28 days of age because very few feces could be collected before weaning at 28 days of age. Ashida H^10^ and Tilg H^12^ showed that dietary fiber is mainly degraded by the gut microbiota. Jumpertz R *et al*^24^ described the associations between nutrient absorption and the gut microbiota in humans, which indicated a possible role of the human gut microbiota in the regulation of nutrient harvest. In previous studies, *Bacteroides* and *Butyrivibrio* were related to protein digestibility in sheep and cows, respectively^25^,^26^. In this study, the apparent digestibility of total tract CF increased as pigs aged (*P*<0.01). Furthermore, no difference in apparent neutral detergent fiber (NDF) digestibility was observed over the whole time period (R = 0.17). Although the apparent NDF digestibility increased from 60 to 90 days of age (*P*<0.01), no significant changes were observed during later growth stages. Additionally, the apparent ADF digestibility increased as the pigs aged (*P*<0.01), but the apparent hemicellulose digestibility showed nearly no change. In fact, the apparent ADF digestibility did not significantly increase from 60 to 90 days of age. Higher apparent ADF digestibility appeared at 150 days of age than that at 60 and 90 days of age (*P*<0.01) (Table 1). Coincidentally, significant differences in the abundance of *Anaeroplasma*, *Campylobacter*, *Clostridium*, *Enterococcus*, *Methanobrevibacter, Nitrosospira*, *Propionibacterium*, *Pseudobutyrivibrio*, *Robinsoniella*, *Staphylococcus* and *Treponema* (Fig. 4, Table 3) were found among the groups and were positively correlated with apparent CF digestibility (*P*<0.05). The bacterial abundances of *Anaeroplasma*, *Campylobacter*, *Caulobacter*, *Cloacibacillus*, *Enterococcus*, *Lactobacillus*, *Methanobrevibacter*, *Nitrosospira*, *Propionibacterium*, *Pseudomonas*, *Robinsoniella*, *Staphylococcus* and *Treponema* differed among the groups and were positively correlated with apparent ADF digestibility (*P*<0.05) (Table 3). Only *Clostridium* was related to the metabolism of dietary fiber^27^, which indicates that the other genera may be associated with apparent CF and ADF digestibility. However, no relationship was found between the microbiota and apparent CP digestibility among the three groups in this study. This may be because protein is mainly digested in the small intestine, as well as because the continual development of digestive organs and their functions improves the apparent nutrient digestibility in pigs^4^,^28^. Additionally, no significant difference was observed in the apparent EE digestibility. Therefore, the relationship between the apparent EE digestibility and the microbiota was not analyzed in this study.

## Conclusion

Both the abundance and diversity of the gut microbiota in pigs increased with increasing age. With increasing age, the proportion of *TM7* and *Tenericutes* increased, whereas the proportion of *Lentisphaerae* and *Synergistetes* decreased. The bacterial abundance of 36 genera changed as the pigs aged. A total of eleven genera, *Anaeroplasma*, *Campylobacter*, *Clostridium*, *Enterococcus*, *Methanobrevibacter, Nitrosospira*, *Propionibacterium*, *Pseudobutyrivibrio*, *Robinsoniella*, *Staphylococcus* and *Treponema*, were correlated with apparent CF digestibility; of these, *Clostridium* is associated with dietary fiber metabolism.

## Materials and Methods

### Animals and sample collection

Fecal and diet samples from 33 healthy Sutai pigs (who did not have disease or diarrhea in the week before sampling) at the ages of 28 (n = 8), 60 (n = 8), 90 (n = 8) and 150 (n = 9) days were randomly collected at the Sutai Pig Breeding Center, Suzhou, China, under similar husbandry conditions. Sutai is a synthetic Chinese breed that is derived from Western Duroc (50%) and Chinese Taihu (50%) after over 18 generations of artificial selection^29^,^30^,^31^. All Sutai pigs from the Sutai Pig Breeding Center were selected using a unified breeding standard. Animals were grouped based on a randomized block experimental design and fed a corn-soybean non-antibiotic diet. Details are provided in the supplementary material describing pig diet ingredients (Table S10A, B). All procedures involving animals were carried out in accordance with the Guide for the Care and Use of Laboratory Animals prepared by the Institutional Animal Care and Use Committee of Nanjing Agricultural University, Nanjing, China. All experimental protocols were approved by the Animal Care and Use Committee of Nanjing Agricultural University, 1999.

Pigs were housed in groups, 8 pigs in a pen. For each sampling (at the ages of 28, 60, 90 and 150 days), all pigs were selected from different pens. Fecal samples were individually collected using sterile 2 ml centrifuge tubes (without any treatment) and plastic bags (approximately 200 g of each fecal sample was fixed on site by mixing with 15 ml 10% sulfuric acid and was kept on ice for transportation) and then stored at −20 °C in the laboratory before DNA extraction and apparent nutrient digestibility assessment.

### DNA extraction, PCR amplification of 16S rDNA, amplicon sequence and sequence data processing

Microbial genomic DNA was extracted from 220 mg of each fecal sample using a TIANamp Stool DNA Kit (Spin Column, Cat. no. DP328) according to the manufacturer's recommendation. Successful DNA isolation was confirmed by agarose gel electrophoresis^23^.

Based on previous comparisons^32^,^33^,^34^, the V4 hypervariable regions of 16S rDNA were PCR amplified from microbial genomic DNA harvested from fecal samples and were used for the remainder of the study. PCR primers flanking the V4 hyper variable region of bacterial 16S rDNA were designed. The barcoded fusion forward primer was 520F 5-AYTGGGYDTAAAGNG-3, and the reverse primer was 802R 5-TACNVGGGTATCTAATCC-3. The PCR conditions were as follows: one pre-denaturation cycle at 94°C for 4 min, 25 cycles of denaturation at 94°C for 30 s, annealing at 50°C for 45 s, and elongation at 72°C for 30 s, and one post-elongation cycle at 72°C for 5 min. The PCR amplicon products were separated on 0.8% agarose gels and extracted from the gels. Only PCR products without primer dimers and contaminant bands were used for sequencing by synthesis. Barcoded V4 amplicons were sequenced using the paired-end method by Illumina MiSeq with a 7-cycle index read. Sequences with an average phred score lower than 30, ambiguous bases, homopolymer runs exceeding 6 bp, primer mismatches, or sequence lengths shorter than 100 bp were removed. Only sequences with an overlap longer than 10 bp and without any mismatch were assembled according to their overlap sequence. Reads that could not be assembled were discarded. Barcode and sequencing primers were trimmed from the assembled sequence^32^.

### Taxonomy classification and statistical analysis

Taxon-dependent analysis was conducted using the Ribosomal Database Project (RDP) classifier^34^. The RDP classifier is a web-based program that assigns 16S rRNA sequences to phylogenetically consistent bacterial taxonomy. OTUs were counted for each sample to express the richness of bacterial species with an identity cutoff of 97%. The OTU abundance of each sample was generated at the genus level. The mean length of all effective bacterial sequences without primers was 223 bp. The abundance count at the genus level was log2 transformed and then normalized as follows: from each log-transformed measure, the arithmetic mean of all transformed values was subtracted, and the difference was divided by the standard deviation of all log-transformed values for a given sample. After this procedure, the abundance profiles for all samples exhibited a mean of 0 and a standard deviation of 1.

A Venn diagram was generated to compare OTUs between groups, and the bacterial community indices applied here included Chao1 and Good's coverage. The bacterial diversity is shown by the number of OTUs. Unweighted clustering was performed using PCoA of UniFrac distance matrices.

### Experimental feeds and chemical analysis

Diet samples were collected in plastic bags and stored at −20°C. Pigs did not receive antibiotics in the feed or for any therapeutic purposes.

For the piglets at 28 days of age and before weaning, measuring the apparent nutrient digestibility from the limited volume of feces was difficult. Fecal samples from pigs at 60, 90 and 150 days of age were dried at 65°C to a constant weight. Fecal and diet samples were ground through a 0.45 mm sieve by a high-speed universal disintegrator before analysis. Acid insoluble ash (AIA) was used as an indigestible marker to assess the digestibility of the dietary components (AOAC 942.05)^35^. Analysis of CF, NDF, ADF and hemicellulose digestibility was carried out using the ANKOM A200 filter bag technique (AOAC 962.09). The CP content was measured based on the Kjeldahl method (AOAC 984.13) using a Kjeltec 8400 analyzer unit (Foss, Sweden), and the EE content was measured using the soxhlet extraction method (AOAC 920.85), which was performed with a soxhlet apparatus.

The contents of different nutrient compositions were calculated as follows: 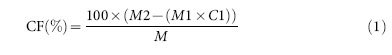
Where: M = Sample weight M1 = Bag tare weight M2 = Weight of organic matter (the loss of weight upon ignition of the bag and fiber) C1 = Ash-corrected blank bag factor (a running average of the loss of weight upon ignition of the blank bag/original blank bag) 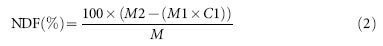


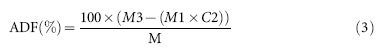

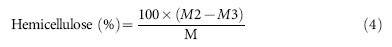
Where: M = Sample weight M1 = Bag tare weight M2 = Weight of organic matter after extraction by neutral detergent M3 = Weight of organic matter after extraction by acid detergent C1 = Ash-corrected blank bag factor (a running average of the loss of weight after extraction of the blank bag/original blank bag) C2 = Ash-corrected blank bag factor (a running average of the loss of weight after extraction of the blank bag/original blank bag)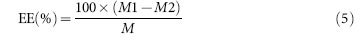 Where: M = Dry sample weight M1 = Filter paper weight before extraction M2 = Filter paper weight after extraction

The digestibility of each sample diet was calculated by the indicator technique^36^according to the equation:
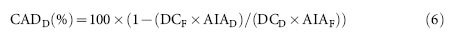
where CAD_D _is the coefficient of the apparent digestibility of dietary components in the assay diet; DC_F_ is the dietary component concentration in feces; AIA_D _is the AIA concentration in the assay diet; DC_D_ is the dietary component concentration in the assay diet; and AIA_F_ is the AIA concentration in feces.

### Data analysis

High quality sequences were uploaded to QIIME for further study^37^. All effective bacterial sequences were compared to the RDP databases using the best hit classification option to classify the abundance count of each taxon^34^,^38^. The sequence length was archived by QIIME. The abundance and diversity indices were generated using Mothur with an OTU identity cutoff of 97% after implementing a pseudo-single linkage algorithm^1,39^. The relationship between the selected taxonomy group (abundant phyla, genera, classes, orders or families), the observed OTUs or the bacterial community index (Chao1) and the apparent nutrient digestibility was calculated using SPSS 13.0 software^39^. For all parameters, data were compared using a one-way analysis of variance (ANOVA) at the end of each bioassay. A mean comparison was performed using Fisher's least significant difference test (LSD) and the Duncan multiple range test with a significance level of *P*<0.05. Finally, Pearson's correlations were used to assess the associations among age, bacterial abundance and apparent nutrient digestibility (*P*<0.05).

## Author Contributions

Conceived and designed the experiments: RHH, PHL, BZ. Performed the experiments: QN, PHL, SSH, HZL, YQZ, XM, SG. Analyzed the data: QN, PHL, BZ, LCH. Contributed reagents/materials/analysis tools: QN, RHH, PHL, XGH, JDH, WJW. Contributed to the writing of the manuscript: QN, PHL, RHH, SWK, BZ. All authors reviewed the manuscript.

## Supplementary Material

Supplementary Information Supplementary information

## Figures and Tables

**Table 1:** The apparent nutrient digestibility in the four groups and Pearson's correlations with age. ^a,b,^* The correlation is significant at a level of 0.05; ^A,B,^** the correlation is significant at a level of 0.01.

Age				
Sample component	60 d	90 d	150 d	Pearson's correlation
CF %	37.05±2.49 ^C^	49.11±3.14^ B^	63.10±1.78 ^A^	0.84 **
NDF %	56.74±2.29 ^B^	69.35±1.48 ^A^	62.43±3.53 ^AB^	0.17
ADF %	28.46±2.71 ^B^	30.53±2.48 ^B^	44.55±2.70 ^A^	0.68 **
Hemicellulose %	74.95±4.19	74.30±3.86	73.93±2.34	−0.03
CP %	73.66±1.14 ^Bb^	77.18±0.90 ^Aa^	77.39±0.68 ^Aa^	0.38 *
EE %	26.83±3.77	20.83±3.78	26.97±2.73	0.07

**Table 2:** Phyla correlated to the apparent fiber digestibility and Pearson's correlation between phyla and apparent fiber digestibility. * The correlation is significant at a level of 0.05; ** the correlation is significant at a level of 0.01.

Phyla	Apparent CF digestibility	Apparent ADF digestibility	Apparent hemicellulose digestibility
	Pearson's correlation	Pearson's correlation	Pearson's correlation
*Euryarchaeota*	−0.054	−0.062	0.448*
*Proteobacteria*	0.508**	0.593**	−0.082
*Spirochaetes*	−0.089	−0.071	0.400*
*Tenericutes*	0.445*	0.301	−0.114
*TM7*	0.516**	0.599**	0.016

**Table 3:** Genera correlated to fiber digestibility and Pearson's correlation between genera and apparent fiber digestibility. * The correlation is significant at a level of 0.05; ** the correlation is significant at a level of 0.01.

Genera	Apparent CF digestibility	Apparent NDF digestibility	Apparent ADF digestibility	Apparent hemicellulose digestibility
	Pearson's correlation	Pearson's correlation	Pearson's correlation	Pearson's correlation
*Anaeroplasma*	0.60**	−0.1	0.48*	−0.18
*Campylobacter*	0.51**	−0.05	0.44*	−0.06
*Caulobacter*	0.3	−0.17	0.44*	−0.12
*Cloacibacillus*	0.3	−0.17	0.55**	−0.23
*Clostridium*	0.45*	−0.03	0.34	−0.06
*Enterococcus*	0.42*	−0.3	0.43*	−0.24
*Janibacter*	0.4	−0.21	0.34	−0.22
*Lactobacillus*	0.12	0.1	0.50*	0.07
*Methanobrevibacter*	0.57**	0.45	0.56**	0.33
*Nitrosospira*	0.43*	0.09	0.49*	−0.01
*Parasporobacterium*	0.22	0.47*	0.12	0.22
*Propionibacterium*	0.50*	0.02	0.46*	0.01
*Pseudobutyrivibrio*	0.51**	−0.14	0.37	0.07
*Pseudomonas*	0.25	−0.05	0.46*	−0.1
*Robinsoniella*	0.61**	0.34	0.65**	0.22
*Staphylococcus*	0.40*	−0.28	0.48*	−0.33
*Sporobacter*	0.32	0.44	0.3	0.19
*Treponema*	0.54**	0.55**	0.54**	0.33
