# Supplementary material for: Dynamic Distribution of the Gut Microbiota and the Relationship with Apparent Crude Fiber Digestibility and Growth Stages in Pigs
Source: Sci Rep. 2015 Apr 21;5:9938. doi: 10.1038/srep09938 (PMC4404679; doi:10.1038/srep09938)
Supplement: Supplementary Information [file srep09938-s1.pdf]

Dynamic Distribution of the Gut Microbiota and the Relationship with Apparent Crude Fiber Digestibility and Growth Stages in Pigs

Qing Niu [1,2+], Pinghua Li [1,2+], Shuaishuai Hao [1,2], Yeqiu Zhang [1,2], Sung Woo Kim [3], Huizhi Li [1,2], Xiang Ma [1,2], Shuo Gao [1,2], Lichun He [1,2], WangJun Wu [1,2], Xuegen Huang [4], Jindi Hua [4], Bo Zhou [1,2*] & Ruihua Huang [1,2*]

[1] Institute of Swine Science, Nanjing Agricultural University, Nanjing, 210095, China, [2] Huaian Academy of Nanjing Agricultural University, Huaian, 223005, China, [3] Department of Animal Science, North Carolina State University, Raleigh, North Carolina, 27695, United States of America, [4] Sutai Pig Breeding Center, Suzhou, 215000, China

* Corresponding author: Ruihua Huang and Bo Zhou. Institute of Swine Science, Nanjing Agricultural University, Nanjing 210095, China. Tel: +86-25-84395362; +86-13814540789; Fax: +86-25-84395314; E-mail: Ruihua Huang, hrh2002@163.com.

+ These authors contributed equally to this work.

Supporting Information

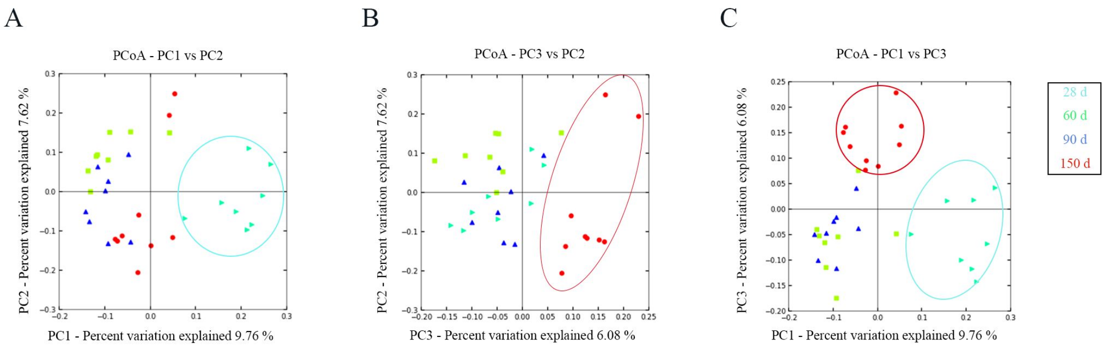

Fig. S1A-C PCoA plots. PCoA of unweighted UniFrac distances for the fecal microbiota at different ages. In the unweighted UniFrac analysis of the fecal samples, the first principal coordinate, explained 9.76% of sample variation, separated groups (28 day of age) from others. The third principal coordinate (6.08% of sample variation) separated groups (150 day of age) from others.

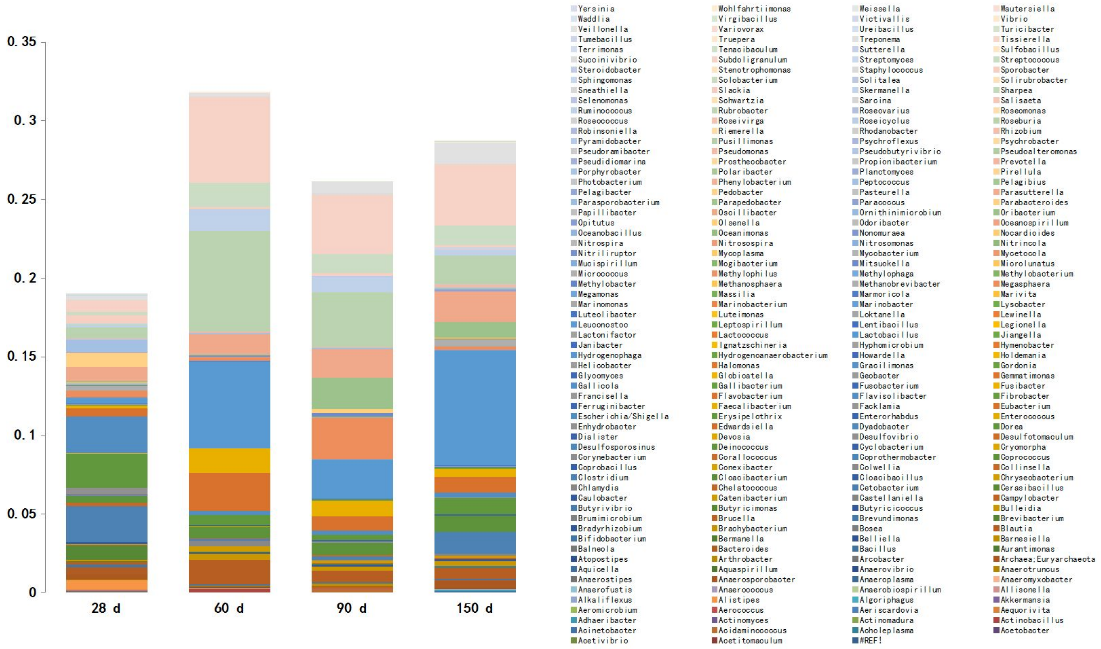

Fig. S2 Relative abundances of distinct genera in four groups. Distribution of the genera as a percentage of the total number of identified 16S rDNA sequences in individual groups.

Table S1 Raw reads, sequences and OTUs from four groups.

Group	Raw reads	High quality sequences	OTUs
28 d	1,532,635	1,003,915	14,306
60 d	1,532,635	822,609	15,051
90 d	1,364,816	964,057	16,329
150 d	1,307,708	1,138,078	17,593

Table S2 Pearson's correlations between age and 5 distinct phyla in four growing phases. * The correlation is significant at a level of 0.05; ** the correlation is significant at a level of 0.01.

**phyla**	**Age**	
	**Pearson's correlation**	***p***
TM7	0.664 **	0.000
Tenericutes	0.658**	0.000
Synergistetes	- 0.495**	0.003
Lentisphaerae	- 0.420*	0.015
Fusobacteria	- 0.327	0.064

Table S3 The relative abundances of 36 distinct genus in four groups were significantly different, and Pearson's correlations with age. ^{a,b,*} The correlation is significant at a level of 0.05; ^{A,B,**} the correlation is significant at a level of 0.01.

Genera	Age				
	28 d	60 d	90 d	150 d	Pearson's correlation
Actinomyces	14.63±4.06 ^{A}	0.25±0.16 ^{B}	0.63±0.38 ^{B}	2.11±1.29 ^{B}	-0.433*
Aeromicrobium	0.88±0.52 ^{B}	0.13±0.13 ^{B}	0.00±0.00 ^{B}	2.78±1.18 ^{A}	0.373*
Akkermansia	70.00±33.99 ^{A}	1.50±0.63 ^{B}	1.38±0.32 ^{B}	2.00±0.67 ^{B}	-0.379*
Anaeroplasma	2.75±0.41 ^{B}	7.75±4.91 ^{B}	7.38±3.42 ^{B}	35.56±6.91 ^{A}	0.669**
Anaerosporobacter	10.00±2.77 ^{b}	17.25±3.98 ^{b}	21.88±6.77	55.00±20.65 ^{a}	0.464**
Atopostipes	0.75±0.25	0.00±0.00 ^{b}	0.63±0.18	2.22±1.15 ^{a}	0.346*
Aurantimonas	1.00±0.73	0.25±0.16 ^{b}	0.63±0.38 ^{b}	9.78±5.69 ^{a}	0.375*
Brevundimonas	19.63±12.17	10.50±3.96 ^{b}	5.50±2.00 ^{b}	115.56±61.08 ^{a}	0.373*
Butyricicoccus	39.88±5.90 ^{b}	103.50±21.53	155.63±43.39 ^{b}	154.89±42.47 ^{b}	0.403*
Caulobacter	0.38±0.26 ^{b}	0.25±0.25 ^{b}	0.50±0.38 ^{b}	2.67±1.22 ^{a}	0.420*
Cloacibacillus	167.13±66.72 ^{A}	2.25±0.37 ^{B}	1.38±0.38 ^{B}	2.56±0.50 ^{B}	-0.436*
Collinsella	264.13±106.97 ^{a}	63.00±13.79 ^{b}	117.75±67.03	37.11±11.60 ^{b}	-0.362*
Deinococcus	0.75±0.41	0.50±0.27 ^{b}	0.13±0.13 ^{b}	2.78±1.27 ^{a}	0.360*
Desulfovibrio	533.63±176.21 ^{A}	10.38±2.88 ^{B}	72.25±44.84 ^{B}	23.00±4.77 ^{B}	-0.464*
Gallibacterium	0.63±0.50 ^{b}	0.25±0.16 ^{b}	0.00±0.00 ^{b}	3.00±1.36 ^{a}	0.391*
Globicatella	1.25±0.41 ^{A}	0.00±0.00 ^{B}	0.13±0.13 ^{B}	0.11±0.11 ^{B}	-0.419*
Gordonia	6.25±5.54	1.00±0.27 ^{b}	0.38±0.18 ^{b}	37.89±20.41 ^{b}	0.370*

Hyphomicrobium	0.50±0.19	0.50±0.38	0.38±0.26 ^{b}	2.00±0.87 ^{a}	0.368*
Lactobacillus	471.88±64.38 ^{b}	5,698.25±1,962.60	2,965.25±1,482.88	9,190.78±4,344.15 ^{a}	0.360*
Lactonifactor	6.50±2.02 ^{A}	0.50±0.27 ^{B}	0.38±0.18 ^{B}	0.56±0.29 ^{B}	-0.472**
Marmoricola	0.38±0.26 ^{b}	0.38±0.26 ^{b}	0.25±0.16 ^{b}	5.44±2.86 ^{a}	0.411*
Methanosphaera	0.00±0.00 ^{b}	0.00±0.00 ^{b}	0.38±0.26	2.89±1.71 ^{a}	0.404*
Nitrosospira	0.50±0.38	0.13±0.13 ^{B}	0.25±0.25 ^{b}	1.22±0.28 ^{Aa}	0.378*
Nocardioides	15.50±10.99	2.88±0.79 ^{b}	1.50±0.53 ^{b}	57.89±27.84 ^{a}	0.360*
Oribacterium	66.38±8.19 ^{B}	62.75±5.81 ^{B}	2,426.13±647.27 ^{b}	1,219.67±491.62 ^{Aa}	0.358*
Paracoccus	22.75±17.33 ^{b}	5.88±2.44 ^{b}	4.25±1.10 ^{b}	151.44±68.04 ^{a}	0.431*
Phenylobacterium	0.63±0.38	1.25±0.73	1.00±0.50	4.22±2.30	0.345*
Pseudobutyrivibrio	5.13±1.06 ^{B}	15.00±4.72 ^{B}	5.25±1.37 ^{B}	56.33±12.46 ^{A}	0.646**
Pseudomonas	38.00±14.63 ^{B}	18.00±4.03 ^{B}	15.63±2.92 ^{B}	204.78±84.47 ^{A}	0.449**
Robinsoniella	0.25±0.25 ^{B}	0.38±0.18 ^{B}	0.38±0.18 ^{B}	5.44±2.15 ^{A}	0.515**
Roseomonas	1.00±0.63 ^{b}	0.13±0.13 ^{b}	0.13±0.13 ^{b}	6.00±2.92 ^{a}	0.404*
Sharpea	109.63±51.54 ^{a}	11.13±3.80 ^{b}	31.13±19.67	11.22±3.49 ^{b}	-0.345*
Solobacterium	1.13±0.40 ^{A}	0.00±0.00 ^{B}	0.13±0.13 ^{B}	0.00±0.00 ^{B}	-0.460**
Sphingomonas	17.75±11.98	3.13±0.77 ^{b}	2.88±0.64 ^{b}	132.78±80.09 ^{a}	0.353*
Treponema	241.00±63.75	1,670.78±923.65	202.50±60.40	803.88±194.05	0.385*
Victivallis	32.13±13.78 ^{A}	3.63±3.34 ^{B}	2.63±0.73 ^{B}	1.22±0.64 ^{B}	-0.420*

37

Table S4 The relative abundances of 12 distinct classes in four groups were significantly different, and Pearson's correlations with age. ^{a,b,*} The correlation is significant at a level of 0.05; ^{A,B,**} the correlation is significant at a level of 0.01.

Classes	Age				
	**28 d**	**60 d**	**90 d**	**150 d**	**Pearson's correlation**
Bacilli	975.25±148.96 ^{b}	7756.88±1923.42	4742.50±1783.75	11379.89±4511.41 ^{a}	0.396*
Bacteroidia	6413.13±1970.41 ^{A}	629.25±100.20 ^{B}	1086.50±187.76 ^{B}	1354.22±305.74 ^{B}	-0.405**
Deltaproteobacteria	589.25±191.77 ^{A}	16.88±5.60 ^{B}	75.25±45.62 ^{B}	36.33±6.55 ^{B}	-0.460**
Elusimicrobia	0.13±0.13 ^{b}	0.13±0.13 ^{b}	1.13±0.61	1.56±0.65 ^{a}	0.419*
Erysipelotrichia	3669.75±1003.60 ^{b}	5951.00±1396.07	5578.13±801.34	8650.56±1912.63 ^{a}	0.414*
Lentisphaeria	36.75±15.10 ^{A}	3.63±3.34 ^{B}	2.88±0.74 ^{B}	1.44±0.78 ^{B}	-0.429*
Mollicutes	4.25±1.05 ^{B}	10.63±4.94 ^{B}	8.50±3.06 ^{B}	37.00±6.41 ^{A}	0.678**
Spirochaetes	186.38±50.53	168.88±59.23	670.38±149.73	1373.11±850.23	0.353*
Subdivision5	5.25±1.05	2.25±0.45	3.13±0.77	18.89±6.18 ^{A}	0.489**
Synergistia	200.75±63.04 ^{A}	2.75±0.41 ^{B}	1.75±0.45 ^{B}	3.11±0.54 ^{B}	-0.496**
TM7_genera_incertae_sedis	15.38±5.11 ^{B}	3.75±0.77 ^{B}	41.13±10.95 ^{B}	320.44±84.44 ^{A}	0.663**
Verrucomicrobiae	78.75±32.83 ^{A}	4.75±1.01 ^{B}	4.00±0.98 ^{B}	7.67±2.03 ^{B}	-0.392*

Table S5 The relative abundances of 17 distinct orders in four groups were significantly different, and Pearson's correlations with age. ^{a,b,*} The correlation is significant at a level of 0.05; ^{A,B,**} the correlation is significant at a level of 0.01.

Orders	Age				
	28 d	60 d	90- d	150 d	Pearson's correlation
Alphaproteobacteria_incertae_sedis	0.13± 0.13 ^{B}	0.50±0.50	0.13±0.13	1.33±0.58 ^{A}	0.353*
Anaeroplasmatales	2.88± 0.77 ^{B}	7.88±4.90	7.00±3.35 ^{B}	35.22±6.88 ^{A}	0.664**
Bacteroidales	6413.13± 1970.41 ^{A}	629.25±100.20 ^{B}	1086.50±187.76 ^{B}	1354.22±305.74 ^{B}	-0.405*
Caulobacterales	21.13± 12.42 ^{b}	13.13±4.61 ^{b}	7.38±2.87 ^{b}	123.67±62.71 ^{a}	0.384*
Desulfovibrionales	581.88± 191.20 ^{A}	10.50±2.89 ^{B}	72.88±45.20 ^{B}	25.78±5.02 ^{B}	-0.465**
Elusimicrobiales	0.13± 0.13 ^{b}	0.13±0.13 ^{b}	1.13±0.61	1.56±0.65 ^{a}	0.419*
Erysipelotrichales	3669.75± 1003.60 ^{b}	5951.00±1396.07	5578.13±801.34	8650.56±1912.63 ^{a}	0.414*
Lactobacillales	882.13±143.88 ^{b}	7673.25±1920.99	4694.75±1776.72	11273.56±4500.22 ^{a}	0.397*
Pseudomonadales	83.38±38.08 ^{A}	39.75±13.06 ^{B}	28.00±5.22 ^{B}	382.44±120.60 ^{B}	0.509**
Rhodobacterales	46.38±29.15 ^{b}	24.00±11.98 ^{b}	11.50±3.59 ^{B}	208.00±87.55 ^{Aa}	0.412*
Sphingomonadales	23.00±15.80	5.75±2.24 ^{b}	4.50±0.98 ^{b}	164.56±95.28 ^{a}	0.362*
Spirochaetales	186.38±50.53	168.88±59.23	670.38±149.73	1373.11±850.23	0.353*
Subdivision5_genera_incertae_sedis	5.25±1.05 ^{B}	2.25±0.45 ^{B}	3.13±0.77 ^{B}	18.89±6.18 ^{A}	0.489**
Synergistales	200.75±63.04 ^{A}	2.75±0.41 ^{B}	1.75±0.45 ^{B}	3.11±0.54 ^{B}	-0.496**
TM7_genera_incertae_sedis	15.38±5.11 ^{B}	3.75±0.77 ^{B}	41.13±10.95 ^{B}	320.44±84.44 ^{A}	0.663**
Verrucomicrobiales	78.75±32.83 ^{A}	4.75±1.01 ^{B}	4.00±0.98 ^{B}	7.67±2.03 ^{B}	-0.392*
Victivallales	36.75±15.10 ^{A}	3.63±3.34 ^{B}	2.88±0.74 ^{B}	1.44±0.78 ^{B}	-0.429*

Table S6 The relative abundances of 8 distinct families in four groups were significantly different, and Pearson's correlations with age. ^{a,b,*} The correlation is significant at a level of 0.05; ^{A,B,**} the correlation is significant at a level of 0.01.

**Families**	**Age**				
	**28 d**	**60 d**	**90 d**	**150 d**	**Pearson's correlation**
Anaeroplasmataceae	39.13±6.42 ^{A}	7.75±4.91 ^{B}	3.38±0.75 ^{B}	6.33±3.03 ^{B}	-0.544**
Deinococcaceae	2.38±1.36 ^{a}	1.25±0.73	0.75±0.41	0.11±0.11 ^{b}	-0.355*
Eubacteriaceae	169.00±36.70 ^{A}	59.00±18.60 ^{B}	30.13±7.64 ^{B}	48.89±10.28 ^{B}	-0.481**
Intrasporangiaceae	105.25±48.76 ^{a}	16.13±12.43 ^{b}	38.75±34.62	2.44±0.88 ^{b}	-0.350*
Subdivision5_genera_incertae_sedis	21.00±6.59 ^{A}	2.25±0.45 ^{B}	4.75±1.11 ^{B}	3.44±0.75 ^{B}	-0.433*
Succinivibrionaceae	80.63±39.08 ^{b}	76.25±24.17 ^{b}	30.88±12.85 ^{B}	196.89±50.86 ^{Aa}	0.385*
TM7_genera_incertae_sedis	358.75±85.33 ^{A}	5.13±1.48 ^{B}	15.25±5.15 ^{B}	37.00±10.50 ^{B}	-0.500**
Veillonellaceae	753.75±342.63 ^{B}	720.13±127.37 ^{B}	413.25±144.94 ^{B}	4308.11±1655.80 ^{A}	0.465**

Table S7A,B Ingredient composition (g/kg) of the experimental diets.

A

**28 d**	
crude protein	≥205
Calcium	7-12
moisture	≤130
crude fiber	≤40
TP	≥6
lysine	≥14
crude ash	≤7
salt	3-8

B

	**60 d**	**90 d**	**150 d**
Corn	690	670	660
Soybean meal	200	200	160
Wheat Bran	20	9	140
Fish meal	20	0	0
Barley	30	0	0
De-mold agent	1	0	0
Vitamin A	2.4×10^{-3}-4.2×10^{-3}	1.2×10^{-3}-1.92×10^{-3}	1.2×10^{-3}-1.92×10^{-3}
Vitamin D3	5×10^{-5}-9×10^{-5}	5×10^{-5}-9×10^{-5}	5×10^{-5}-9×10^{-5}

Vitamin E	$\geq 5\times10^{-3}$	$\geq 0.45\times10^{-3}$	$\geq 0.45\times10^{-3}$
Vitamin K3	4.5×10^{-5}-8.5×10^{-5}	4.5×10^{-5}-8.5×10^{-5}	3.5×10^{-5}-6.5×10^{-5}
Vitamin B1	$\geq 5\times10^{-5}$	$\geq 4.5\times10^{-5}$	$\geq 4.5\times10^{-5}$
Vitamin B2	$\geq 1.3\times10^{-5}$	$\geq 1.1\times10^{-5}$	$\geq 1.1\times10^{-5}$
Vitamin B6	$\geq 7.5\times10^{-5}$	$\geq 6.5\times10^{-5}$	$\geq 6.5\times10^{-5}$
Vitamin B12	$\geq 5\times10^{-7}$	$\geq 4\times10^{-7}$	$\geq 4\times10^{-7}$
Niacin	$\geq 7.5\times10^{-4}$	$\geq 0.65\times10^{-4}$	$\geq 0.65\times10^{-4}$
Calcium Pantothenate	$\geq 3\times10^{-4}$	$\geq 0.28\times10^{-4}$	$\geq 0.28\times10^{-4}$
Folat	$\geq 2.5\times10^{-5}$	$\geq 2.3\times10^{-5}$	$\geq 2.3\times10^{-5}$
bBiotin	$\geq 2.5\times10^{-5}$	$\geq 2.3\times10^{-5}$	$\geq 2.3\times10^{-5}$
Choline Chloride	$\geq 7\times10^{-3}$	$\geq 7\times10^{-3}$	$\geq 6.5\times1^{0-3}$
copper	3×10^{-3}-4.5×10^{-3}	2.75×10^{-3}-3.75×10^{-3}	5×10^{-4}-8.75×10^{-4}
Iron	3×10^{-3}-4×10^{-3}	3×10^{-3}-5×10^{-3}	3×10^{-3}-5×10^{-3}
Manganese	1.2×10^{-3}-2×10^{-3}	1.2×10^{-3}-2×10^{-3}	1.2×10^{-3}-2×10^{-3}
Zinc	2.5×10^{-3}-3.75×10^{-3}	2.5×10^{-3}-3.75×10^{-3}	2.5×10^{-3}-3.75×10^{-3}
Iodine	1.7×10^{-5}-3.3×10^{-5}	1.7×10^{-5}-3.3×10^{-5}	1.7×10^{-5}-3.3×10^{-5}
Selenium	7×10^{-6}-1.2×10^{-6}	7×10^{-6}-1.2×10^{-6}	7×10^{-6}-1.2×10^{-6}
Calcium	4.8-6.4	4.4-6	4-5.6
Total Phosphorus (TP)	≥0.1	≥0.1	≥0.56
Salt	2.8-4.4%	2.8-4.4%	2.8-4.4%
Moisture	≤4	≤4	≤4
